# Evaluation of serum creatine phosphokinase in diagnosis of tubal ectopic pregnancy compared with intrauterine pregnancy and threatened abortion

**Published:** 2012-07

**Authors:** Maryam Asgharnia, Roya Faraji, Fariba Mirblouk, Zahra Atrkar Roshan, Ayda Parvizi

**Affiliations:** 1 *Reproductive Health Research Center, Department of Obstetrics and Gynecology, Guilan University of Medical Sciences, Guilan, Iran.*; 2 *Department of Biostatistics, Guilan University of Medical Sciences, Guilan, Iran.*

**Keywords:** *Creatine phosphokinase*, *Tubal ectopic pregnancy*, *Threatened abortion*, *Intrautine pregnancy*

## Abstract

**Background:** Vaginal sonograghy and serial -hCG are the most common diagnostic methods for ectopic pregnancy but about 50% of cases are initially misdiagnosed. In tubal pregnancy the zygote lies next to the muscular layer, and this invasion causes an increase in creatine phosphokinase (CPK) in blood.

**Objective: **assessment of CPK and its isoenzyme CPK-MB as a diagnostic marker for tubal pregnancy.

**Materials and Methods:** In this case-control study, 111 women between 16-40 years in first-trimester pregnancy admitted to emergency ward of Rasht Alzahra hospital with abdominal pain or vaginal bleeding were included and according to sonography and βhCG divided into 3 groups (N=37): tubal pregnancy (1), threatened abortion (2) and normal pregnancy (3). Blood samples were taken for totalCPK and CPK-MB before any invasive procedure.

**Results:** Mean total CPK level were 96.27±63.9 u/lit (group 1), 55.37±14.1 u/lit (group 2) and 48.94±19.2 u/lit (group 3) and was significantly higher in tubal pregnancy compared to other groups. Mean CPK-MB levels in 3 groups were 15.62±5.2 u/lit, 17.32±6.9 u/lit, and 15.1±4.7 u/lit, respectively which was not significant.

**Conclusion:** It seems that determination of total CPK can enhance the diagnostic value of tubal pregnancy.

## Introduction

In ectopic pregnancy (EP) the trophoblast implants outside the uterine cavity and this implantation happens in more than 95% of cases in the fallopian tubes (1). In last years the incidence of EP has been rising because of the growing incidence of pelvic inflammatory disease, fertility drugs and pelvic surgery (2). Transvaginal sonography and serial βhCG are the most common diagnostic methods for EP but 50% of cases are initially misdiagnosed (3).

In fact, despite the advances in ultrasound a recent series reported that 48-82% of all patients presenting with abdominal pain and/or vaginal bleeding in the first trimester had an equivocal ultrasound when the quantitive β-hCG was below 1000 mIU/dl. This subgroup of patients in particular cannot be accidently evaluated and may benefit most from a serum marker that is rapidly available and useful in the early diagnosis of tubal pregnancy (2).

Clinical symptoms in EP can be similar to non-EP condition thus there is need to searching for some new diagnostic tools. In a new article, Creatinephosphokinase (CPK) has been suggested as a new diagnostic criteria in EP (4). The fallopian tube lacks a submucosal layer, so in tubal ectopic pregnancy, the zygote lies next to the muscular layer and this invasion causes on increase in CPK level as a marker of smooth muscle injury (5, 6). 

Three distinct isoenzyme forms of CPK have been identified, namely, CPK-MM, MB and BB (M: muscle- B: brain) (7). Lavie *et al* (8) were the first to report that measurement of total CPK levels was both sensitive and specific for the diagnosis of EP. This finding was recently confirmed (2). In another study it was found that serum creatinekinase may help in discriminating ruptured from ruptured EP, whereas it is not useful for the primary diagnosis of ectopic gestation (9). Others have shown that, although women with EP tend to have higher CPK levels, a significant overlap of values, limits the diagnostic value of CPK measurements (10-14). 

Also in one recent study the researcher suggested CPK is an indicator for predicting treatment outcome. In their study CPK level was significantly higher in women who successfully treated for Ectopic pregnancy with only a single injection of methotrexate (15). It must be mentioned that in most of previous studies total CPK levels were measured except for one (7) in which CPK MM and MB levels estimated and found that women with EP had significantly higher CPK and significantly lower CPK-MB relative ratio.

According to controversial results of previous studies the current study was designed to further evaluation the diagnostic value of total CPK in ectopic pregnancy and to evaluate the possible discriminatory ability of its isoenzymes; because in Iran, we measure CPK-total and CPK-MB. (CPK MM can’t be measured in Iran). 

## Materials and methods

In this case-control study, 111 (range 16-40 years) first-trimester pregnant women admitted to emergency ward of Rasht Alzahra Hospital with lower abdominal pain and/or vaginal bleeding were included (from September 2009 to February 2010). 

According to vaginal Ultrasonography and serial βhCG, patients divided into 3 groups with final diagnosis, each group consisted of 37 patients: 1) tubal ectopic pregnancy 2) threatened abortion 3) normal intra-uterine pregnancy (Nl IUP). Patients followed up longitudinally to establish the diagnosis. 

To limit confounding factors, we identified and excluded patients with a recent history of surgery, major truma, chest pain, CNS disorders, hypothyrodism, myopathy or intramuscular injection. Blood samples were taken by routine verinpucture (for total and MB CPK) before any invasive procedure. CPK-total and CPK-MB were measured by photometric pars-azmun kit at 37^o^C the upper reference of total CPK for women was 170 u/Lit at 37^o^C. This study was done with financial support of Vice chancellor of research Guilan University of Medical Sciences.

**Statistical analysis**

Data gathered in special checklists and finally analyzed with SPSS software. The categorical outcome variables compared with One Way ANOVA test. The statistical significance was set at 0.05 levels.

## Results

**Figure 1:**
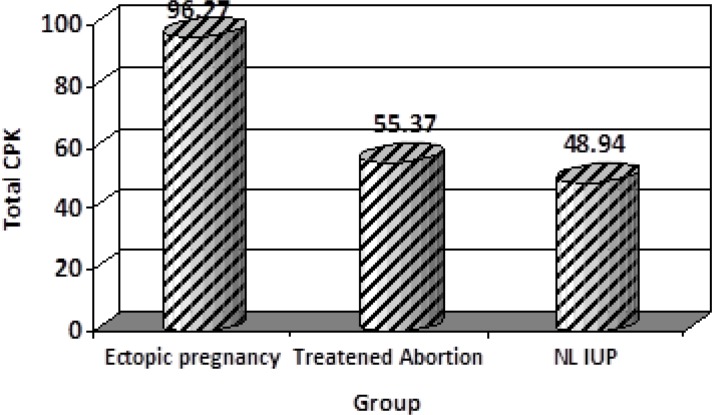
The mean level of total CPK in three groups

In cases the mean of age was 27.6±5.8 (range 16-40 years). Most of them were in age group of 25-29 (34.2%). We had 111 cases in 3 groups; each group consisted of 37 patients. From 37 patients in EP group, one case had fetal heart rate in sonograghy. We had 5 cases of ruptured EP in this group. Most of EP patients received Methotraxate therapy [26 patients of EP groups (70.2%)], 4 patients (10.8%) managed by laparoscopy and for 7 (19%) of them laparatomy was performed. The mean of total CPK are shown in figure 1, which was 96.27±63.9 u/lit for EP, 55.37±14.1 u/lit for threatened abortion and 48.94±19.2 u/lit for intaurine pregnancy.

In one way ANOVA test the mean level of total CPK was significantly higher in tubal pregnancy compared to other groups (p<0.0001) (Figure 1). The mean levels for CPK-MB was 15.62±5.2 u/lit for EP, 17.32±6.9 u/lit for threatened abortion and 15.1±4.7 u/lit for NL pregnancy. The difference between 3 groups for CPK-MB was not significant (Table I). In Ep group we had 5 cases of ruptured EP, which could show the mean level higher than real. To diminish this confounding effect we analyzed data again after omitting these cases (Table II). Total CPK had still a significant difference. The mean total CPK in Ep was 96.27 u/lit and β-hCG level in EP was 6574.405 u/lit, the correlation of CPK with βhCG in EP group was 107 (p=0.53). We could not find any significant correlation between CPK and βhCG level, in EP group.

**Table I:** The mean level of CPK-MB in three groups

**Groups (n=37)**	**Level of CPK-MB**	**Min**	**Max**	**p-value**
Ectopic pregnancy	15.62 ± 5.2	5	30	0.219 (NS)
Threatened abortion	17.32 ± 6.9	8	33	
NL IUP	15.1 ± 4.7	6	26	

Results are presented as the mean±SD.

**Table II:** The mean level of total CPK after omitting ruptured EP

**Groups (n=37)**	**Total CPK after omitting ruptured EP (u/lit)**	**Min**	**Max**	**p-value**
Unruptured ectopic pregnancy (n=32)	86.40 ± 51.6	34	330	0.0001
Threatened abortion	55.37 ± 14.1	35	92	
NL IUP	48.94 ± 19.2	20	85

Results are presented as the mean±SD.

## Discussion

Ectopic pregnancy is a relatively common complication, which can be fatal, if not promptly diagnosed. Threatened abortion is another, more benign cause of abdominal pain, which must be distinguished from EP. Therefore it is important to have reliable predictors in the differential diagnosis of these two conditions (7). 

In the current study, total CPK levels were significantly increased in women with EP compared with both women with miscarriage and those with normal gestation (Table I). This difference, in accordance with the result of previous studies (6-9) was expected, because fetal invasion into the tubal smooth muscle layer results in tissue injury, which has been known to raise the concentration of serum CPK. In Soundravally study CPK level was higher in isthmica tubal pregnancies and ruptured ectopic pregnancies. 

It is likely that as tubal pregnancies grows and progresses towards rupture, then serum CPK concentrations are increased thus they concluded this hasn’t proven to be a clinically useful discriminator (1). Katsikis* et al* (7) studied 40 women with EP, 20 with IUP and 20 with abortive gestation and measured total CPK, CPK MM and CPK MB at the time of presentation and 24 hours after surgery and showed that women with EP had significantly higher total CPK and a significantly decreased CPK-MB relative ratio compared with other groups.

In our study, we couldn’t find any significant difference in CPK-MB level in 3 groups. Saha *et al* (16) studied 20 women with EP and 20 women with NL pregnancy in a case-control study. Total CPK level were found to be significantly higher in EP group (34.15±1.17 U/L) compared to the controls (18.72±1.25), which was in accordance of our study. Lavie *et al* (8) enrolled 3 groups of 17 patients for EP, abortion and NL IUP. CPK level was >45 U/Lit in all patients with tubal pregnancy, significantly higher than the level in patients of other groups. Birkhahan (2), Duncan (10), Kurzel (17) and Chandra (18) reported the same results. But on the other hand we have some articles which refused these results. Vitoratos *et al* (5) selected 10 patients with asymptotic tubal pregnancy 11 with symptomatic tubal pregnancy, 20 with NL IUP and 15 with threatened abortion. No significant difference of total CPK levels was observed between groups. 

Birkhan *et al* (6) assessed 278 patients (61with EP-317 with non-Ep) and reported that serum Creatine Phoshokinase, smooth muscle heavy-chain myosin and myoglubin cannot be useful marker for EP. Korhonen (11) and Vandermolen (12) reported the same issues. Develioglu found that they had significant difference in the level of CPK between ampullary and isthmic position of ectopic pregnancy thus this variable may have affected their results (9). The difference between studies can be because of different gestational ages. Gestational location of EP and the degree of tubal distention can be another reason but Kurzel reported that these two items won’t affect the CPK level. 

One possible explanation is that the serum biomarkers often don’t follow a steady pattern over a normal gestation, also all subjects must matched for gestational age because if the subject didn’t be matched for gestational age large differences could be seen within the same group another explanation differing results may be due to artifact of different methods for identifications and the reagent use to detect them (19). All patients in our study were admitted in hospital but the results may differ in patients admitted or patients who are not. In our study the correlation between βhCG and total CPK analyzed which was never done before. We can say determination of total CPK in combination with TVS and serial βhCG can enhance the diagnostic value of Ectopic tubal pregnancy.

## Conclusion

Large-scale prospective studies are needed for better evaluation and to determine a cut-off point for CPK.
